# Antigen-specific immunotherapy of autoimmune and allergic diseases

**DOI:** 10.1016/j.coi.2010.08.006

**Published:** 2010-10

**Authors:** Catherine A Sabatos-Peyton, Johan Verhagen, David C Wraith

**Affiliations:** School of Cellular and Molecular Medicine, University of Bristol, Medical Sciences Building, University Walk, Bristol, BS8 1TD, UK

## Abstract

Nearly a century has passed since the first report describing antigen-specific immunotherapy (antigen-SIT) was published. Research into the use of antigen-SIT in the treatment of both allergic and autoimmune disease has increased dramatically since, although its mechanism of action is only slowly being unravelled. It is clear though, from recent studies, that success of antigen-SIT depends on the induction of regulatory T (T reg) cell subsets that recognise potentially disease-inducing epitopes. The major challenge remaining for the widespread use of antigen-SIT is to safely administer high doses of immunodominant and potentially pathogenic epitopes in a manner that induces T cell tolerance rather than activation. This review illustrates that intelligent design of treatment agents and strategies can lead to the development of safe and effective antigen-SIT.

## Introduction

Current treatments for allergic and autoimmune disease treat disease symptoms or depend on non-specific immune suppression. Treatment would be improved greatly by targeting the fundamental cause of the disease, that is the loss of tolerance to an otherwise innocuous antigen in allergy or self-antigen in autoimmune disease (AID). Much has been learned about the mechanisms of peripheral tolerance in recent years. We now appreciate that antigen presenting cells (APC) may be either immunogenic or tolerogenic, depending on their location, environmental cues and activation state [1]. Furthermore, it is clear that both FoxP3^+^ and FoxP3^−^ cells with regulatory properties can be induced with specific antigen. This, therefore, provides two guiding principles for the design of antigen-specific immunotherapeutic strategies. First, antigen should be directed to tolerogenic rather than immunogenic APC; secondly, administration of antigen should lead to the induction of regulatory T cells.

## Allergen-specific immunotherapy

The application of allergen-SIT has become increasingly popular since first reported by Leonard Noon in 1911. Subcutaneous (s.c.) or oral/sublingual administration of allergens has been used for the successful treatment of a wide range of allergies including those to bee venom [2], cow's milk [3], peanut [4] or birch pollen [5]. Typically, this starts with a build-up day where the maximum tolerated dose is determined. This dose is then gradually escalated over a period of approximately two months to a high maintenance dose, which is administered regularly for months to years. By escalating the treatment dose, a maintenance dose can be reached that is far higher than the maximum tolerated dose at onset, with limited adverse effects. Studies have shown that optimal results are achieved using the highest tolerable maintenance dose [6] or a high cumulative dose, that is long-term treatment [7]. To further prevent adverse effects and increase efficacy, a wide range of novel therapeutic strategies have been employed, including the use of non-IgE binding allergen derivatives, adjuvants, alternative routes of administration, fusion proteins, allergen-encoding cDNA and peptides that represent T cell epitopes (reviewed in [8]). Hypoallergenic peptides, in particular, are an increasingly popular alternative to whole proteins and have proven successful in animal models and human trials [9,10,11^••^].

Progress in the development of allergen-SIT has been hindered by a lack of understanding of the underlying immunological mechanisms. In recent years, it has become clear that the ratio of allergen-specific T cells secreting distinct cytokines plays a crucial role in the onset and cessation of allergic diseases. First, it is important to realise that allergic and non-allergic individuals recognise the same T cell epitopes of common allergens [12] and that only the frequency of different subsets of CD4^+^ T cells specific for these epitopes differs. It is now clear that the balance between effector T cell populations on the one hand and IL-10-secreting, suppressive T cells on the other makes the difference between an atopic or healthy immune response. In atopic individuals, the highest proportion of T cells recognising common environmental allergens are IL-4-secreting T helper (Th) 2 cells, whereas IL-10-secreting T cells prevail in healthy individuals [13]. The importance of the dominance of the IL-10-secreting T cell population for a healthy immune response to allergens is elegantly demonstrated in the case of beekeepers [14^••^]. During the season, beekeepers are stung frequently, thus receiving repetitive high doses of allergen. Remarkably, their immune response to the venom skews rapidly, with a dramatic shift in the dominant T cell subtype towards IL-10-secreting cells. This induction of IL-10-secreting cells has now also been shown to be a dominant feature of successful allergen-SIT in a range of allergies (Table 1) [4,5,11^••^,15,16]. Allergen-SIT is successful at any age but early treatment of a single allergy may prevent epitope spreading and hence limit the atopic march in later life [17].

## Autoantigen-specific immunotherapy

The use of SIT for AID has lagged behind SIT for allergy. This may be because AIDs are more heterogeneous than allergic diseases; the disease-initiating or target antigen may not be known; and/or the immune pathogenesis of AID is associated with epitope spreading [18] and substantial tissue damage may have occurred before an effective diagnosis has been made. Effective SIT for AID will, therefore, require the induction of cells capable of ‘bystander’ regulation or suppression at the earliest stage of disease [19,20].

First attempts at SIT, in diseases such as multiple sclerosis (MS), were not successful [21–23]. Weiner and colleagues extended these studies by testing the phenomenon of mucosal tolerisation in various experimental models [24]. This was universally effective and revealed that a relatively low dose of antigen, delivered by the oral route, would induce ‘bystander suppression’ whereby the administration of antigen A would induce cells capable of suppressing responses to antigens B and C. Clinical trials proved that oral tolerance induction is safe but not as effective as expected from studies in animal models.

The administration of self-antigen via plasmid DNA is an attractive approach since co-expression of cytokines and immune modulators can be used to enhance mechanisms of tolerance induction. Early examples of DNA vaccination in AID models produced conflicting results; disease could be either suppressed [25] or enhanced [26,27], depending on the disease model or antigen expressed. Various approaches have been taken to enhance tolerance induction. CpG motifs in plasmid DNA contribute to the Th1 response; this is reduced by co-administration of an oligonucleotide expressing GpG in place of CpG [28]. A DNA vaccine encoding myelin basic protein, with CpG motifs replaced by GpG, was recently tested in MS patients [29^•^]. Treatment with a 0.5 mg dose of DNA resulted in the reduction of new lesions in the CNS, coinciding with a decrease in the Th1 response to myelin antigens. Co-expression of cytokines designed to reduce the Th1 response to antigen enhanced the efficacy of tolerogenic DNA vaccination in both EAE [30] and type I diabetes (T1D) models [31]. An intriguing, novel approach involves the introduction of the microRNA miR-142 into the antigen-expressing vector [32^••^]. The microRNA suppressed antigen expression in professional APC and led to the induction of antigen-specific FoxP3^+^ cells in the liver. Expression of antigen in the liver generally enhances the generation of induced FoxP3^+^ Treg cells and has proven effective in models of uveitis and MS [33^•^,34^•^].

Although antigenic protein therapy has been successful in pre-clinical models, the approach has not translated well into the clinic. Nevertheless, there has been success in the use of an alum-based islet-antigen vaccine. GAD-alum treatment led to the preservation of residual insulin secretion in patients with recent-onset T1D [35^•^]. One approach, designed to improve the safety of self-antigen delivery in AID, involves the coupling of intact antigen to APC using chemical fixatives [36]. This is based on the concept that fixation promotes a tolerogenic response to the APC. A systematic study of this approach, however, revealed that efficacy is not dependent on the delivery of antigen on autologous cells; in fact, cells may be replaced with antigen-coated beads [37]. This implies that the fixed cells themselves are not directly involved in tolerance induction; rather, they carry intact antigen to tolerogenic APC for processing and presentation. A phase I trial of fixed autologous peripheral blood leukocytes coupled with a cocktail of seven encephalitogenic myelin peptides is underway in early relapsing-remitting MS patients [38].

The alternative to coupling antigen to APC is simply to administer soluble peptide via a tolerogenic route. Much has been learned about the nature of the peptide, dose, route of delivery and timing. The peptide must mimic the naturally processed antigen when bound to major histocompatibility complex (MHC) [39]; post-translational modification of the peptide may be required [40], and treatment must be initiated as soon as possible following definite diagnosis (e.g., a single peptide could suppress diabetes at an early stage of disease while treatment of late stage disease required administration of a combination of peptides [41]). One approach to improving the safety and efficacy of peptide therapy has included the development of recombinant MHC–peptide complexes. Treatment with sIAg7–pGAD65 complexes effectively blocked the development of diabetes in the NOD mouse; suppression was dependent on induction of islet-cell-specific IL-10-secreting CD4^+^ T cells [42]. Similarly, treatment with a single recombinant MHC–peptide complex could reverse EAE through induction of IL-10-secreting regulatory cells [43]. Clinical trials of peptide therapy have shown promising results in a range of AID (Table 2) with various routes of administration and dosing schedules under investigation. Importantly, as in many pre-clinical models, IL-10 is frequently associated with effective peptide therapy.

## The importance of antigen dose and IL-10 production for the success of antigen-SIT

**Figure 1:**
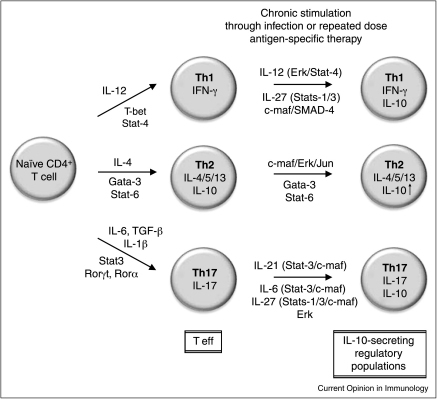
Self-regulatory properties of effector Th populations through chronic/repetitive stimulation. Differentiation of naïve CD4^+^ T cells into effector Th1, Th2 and Th17 populations is well established. Recent evidence from viral-infection and helminth-infection models, as well as numerous allergen-specific and autoantigen-specific peptide therapy trials, suggests that upregulation of IL-10 occurs during chronic or repetitive stimulation and can serve as a self-limiting mechanism [43,44^•^]. Shown are the potential cytokines/transcription factors/signalling molecules that mediate the switch from effector T (T eff) to IL-10-secreting regulatory populations. Note that Th2 cells produce IL-10 upon initial differentiation, and while evidence does suggest that IL-10 helps host survival during helminth infection [44^•^], whether this is a general feature of well-differentiated Th2 cells remains unclear.

**Figure 2:**
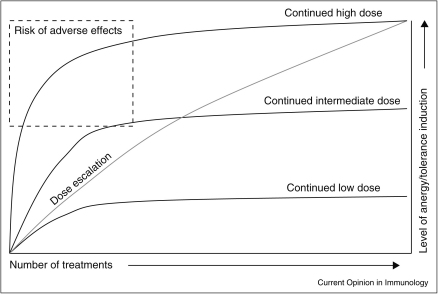
Dosing strategy for antigen-SIT. Repeated administration of high-dose/affinity antigen has the potential to induce the highest level of anergy and tolerance, but the first few treatments may induce acute and sometimes severe side effects. Low-dose antigen does not carry a risk of side effects, nor does it induce robust tolerance. The low dose does, however, allow for the gradual increase of the dose without the adverse effects normally associated with the higher dose.

IL-10 secretion is a common self-regulatory property for the major CD4^+^ T helper subsets, with Th1, Th2, Th9 and Th17 cells all shown to secrete IL-10 in the face of chronic exposure to antigen (reviewed in [44^•^,45]). Both allergen-SIT and autoantigen-SIT exploit this natural IL-10-secreting phenotype of highly differentiated effector cells with repeated exposure to high-dose antigen converting effector T cells to IL-10-secreting regulatory populations (Figure 1). The greatest hazard of high-dose peptide-specific therapy, however, is a harmful immune response due to the initial burst of cell activation with subsequent proliferation and excessive cytokine release. This became evident in trials of altered peptide ligand (APL) therapy in MS. Treatment was terminated when it became evident that an allergic response to the peptide had been induced at the highest dose [46,47]. Importantly, this problem was not observed at lower doses of peptide. We have recently shown, using peptide analogues, that anergy, suppression and IL-10 secretion are dose or affinity dependent, with lower signal strength leading to anergy and higher signal strength driving IL-10 secretion and effective regulation of the inflammatory immune response [48^•^]. Combined with the body of knowledge from allergen-SIT, these data suggest that initiating treatment with lower doses and building to the highest maintenance dose allows the full benefit of tolerance induction while also protecting the recipient from harmful side effects. We propose, therefore, that for antigen-SIT in both allergy and AID, a stepwise approach, with dose escalation activating T cells through increasing strength of signal to a higher maintenance dose, will induce tolerogenic IL-10-secreting cells, capable of suppressing the effector properties of their initiating population (Figure 2).

Some studies suggest that IL-10 secretion is a transitory property of highly stimulated cells, dependent on tissue-specific environmental cues for its maintenance, while others suggest that the IL-10 locus can be genetically modified in terminal differentiation. Recent evidence has shown that sustained high-dose TCR signalling and high levels of IL-12 were required for the induction and maintenance of IL-10 secretion in Th1 cells, through sustained ERK1 and ERK2 MAP kinase phosphorylation [49^••^]. However, for Th2 cells, epigenetic modification of chromatin at the IL-10 locus has been demonstrated [50,51]. Thus, the mechanistic details of IL-10 regulation in T cells and the crucial question of whether IL-10 secretion can be imprinted remain open.

## Conclusion

This review has highlighted recent advances in specific immunotherapy for allergic and autoimmune disease. One overriding conclusion is that regulatory mechanisms involving IL-10 are important for effective therapy. Our belief is that basic research should focus on means to target tolerogenic APC; to promote, for example through the use of appropriate adjuvants, secretion of IL-10 from both APC and T cells; and to investigate the mechanisms of dose escalation tolerance. Future clinical trials should focus on patient groups that are most likely to benefit from the treatment, for example major changes should not be expected in advanced stages of disease [38]; no SIT trial should be undertaken without detailed investigation of immunological changes arising from the treatment; and intervention should be undertaken as early after diagnosis as possible. Finally, the dose of antigen required for SIT is the most critical consideration; dose escalation should allow for a safe increase in dose until an effective, tolerogenic dose is achieved.

## References and recommended reading

Papers of particular interest, published within the period of review, have been highlighted as:

• of special interest
•• of outstanding interest

## Figures and Tables

**Table 1:** Studies demonstrating the importance of IL-10 induction for successful allergen-SIT.

Allergen	Treatment	Patients	Outcome	Ref.
Peanut	Titrated oral administration of peanut protein up to 1800 mg (total 36 months)	29	Reduction in IgE; increased IgG4; increase in IL-10, IL-5, IFN-γ and TNF-α. Increase in antigen-specific FoxP3^+^ cells until 12 months	[4]
Birch pollen	Incremental weekly doses of s.c. standard quality birch pollen allergen up to 100,000 units, followed by monthly maintenance dose	13	Increase in antigen-specific IL-10-secreting cells; increase in allergen-specific IgG antibodies	[5]
Fel d 1 (cat)	Asthma patients received intradermal (i.d.) fel d 1 peptides in increments up to a 90 μg total over two weeks	16	Reduced late reaction to cat dander; increased IL-10, with linked epitope suppression	[11^••^,15]
Japanese cedar pollen	Sublingual application of a pool of 7 Cry j 1 and 2 derived peptides	75	Increase in IL-10-secreting regulatory cells; reduction in allergy symptoms to cedar pollen and other allergens	[16,17]

**Table 2:** Studies demonstrating the efficacy of self-antigenic peptide SIT in autoimmune diseases.

AID	Treatment	Patients	Outcome	Ref.
Rheumatoid Arthritis	DNAJP1 peptide peroral, 25 mg/day over six months	160	Immune deviation from TNF-α to IL-10; combination of peptide and hydroxychloroquine most effective	[52]
Systemic Lupus Erythematosus	Three s.c. doses of spliceosomal peptide P140 at two-week intervals	20	Anti-dsDNA antibody levels reduced in 200 μg group	[53]
T1D	Three i.d. doses of proinsulin peptide at monthly intervals	48	Increase of IL-10-secreting T cells in patients receiving 10 μg dose	[54]
T1D	DiaPep 277, hsp60 peptide, range of doses around 1 mg	>300	IL-10 production in response to therapy associated with preservation of C-peptide	[55,56]
Primary Progressive MS/Secondary Progressive MS	MBP8298, 500 mg i.v. every six months	32	Reduction in CSF anti-MBP. Delay to progression in DR2/DR4 subgroup	[57]
